# Device modeling of two-steps oxygen anneal-based submicron InGaZnO back-end-of-line field-effect transistor enabling short-channel effects suppression

**DOI:** 10.1038/s41598-022-23951-x

**Published:** 2022-11-12

**Authors:** Donguk Kim, Je-Hyuk Kim, Woo Sik Choi, Tae Jun Yang, Jun Tae Jang, Attilio Belmonte, Nouredine Rassoul, Subhali Subhechha, Romain Delhougne, Gouri Sankar Kar, Wonsok Lee, Min Hee Cho, Daewon Ha, Dae Hwan Kim

**Affiliations:** 1 School of Electrical Engineering, Kookmin University, Seoul, 02707 Republic of Korea; 2 imec, Kapeldreef 75, 3001 Leuven, Belgium; 3 Advanced Device Research Lab, Semiconductor R&D Center, Samsung Electronics Co., Ltd., Gyeonggi-do, Hwaseong-si, 18448 Republic of Korea

**Keywords:** Engineering, Electrical and electronic engineering

## Abstract

Amorphous oxide semiconductor (AOS) field-effect transistors (FETs) have been integrated with complementary metal-oxide-semiconductor (CMOS) circuitry in the back end of line (BEOL) CMOS process; they are promising devices creating new and various functionalities. Therefore, it is urgent to understand the physics determining their scalability and establish a physics-based model for a robust device design of AOS BEOL FETs. However, the advantage emphasized to date has been mainly an ultralow leakage current of these devices. A device modeling that comprehensively optimizes the threshold voltage (*V*_T_), the short-channel effect (SCE), the subthreshold swing (SS), and the field-effect mobility (µ_FE_) of short-channel AOS FETs has been rarely reported. In this study, the device modeling of two-steps oxygen anneal-based submicron indium-gallium-zinc-oxide (IGZO) BEOL FET enabling short-channel effects suppression is proposed and experimentally demonstrated. Both the process parameters determining the SCE and the device physics related to the SCE are elucidated through our modeling and a technology computer-aided design (TCAD) simulation. In addition, the procedure of extracting the model parameters is concretely supplied. Noticeably, the proposed device model and simulation framework reproduce all of the measured current–voltage (*I–V*), *V*_T_ roll-off, and drain-induced barrier lowering (DIBL) characteristics according to the changes in the oxygen (O) partial pressure during the deposition of IGZO film, device structure, and channel length. Moreover, the results of an analysis based on the proposed model and the extracted parameters indicate that the SCE of submicron AOS FETs is effectively suppressed when the locally high oxygen-concentration region is used. Applying the two-step oxygen annealing to the double-gate (DG) FET can form this region, the beneficial effect of which is also proven through experimental results; the immunity to SCE is improved as the O-content controlled according to the partial O pressure during oxygen annealing increases. Furthermore, it is found that the essential factors in the device optimization are the subgap density of states (DOS), the oxygen content-dependent diffusion length of either the oxygen vacancy (*V*_O_) or O, and the separation between the top-gate edge and the source-drain contact hole. Our modeling and simulation results make it feasible to comprehensively optimize the device characteristic parameters, such as *V*_T_, SCE, SS, and µ_FE_, of the submicron AOS BEOL FETs by independently controlling the lateral profile of the concentrations of *V*_O_ and O in two-step oxygen anneal process.

## Introduction

Amorphous oxide semiconductor (AOS) field-effect transistors (FETs) are successfully utilized in the backplane panels of commercial display products, such as organic light-emitting diode displays and liquid–crystal displays^1,2^. Their advantages include higher mobility than amorphous Si, good large-area uniformity, and a low process temperature. Furthermore, AOS FETs are promising candidates for the back end of line (BEOL) FETs; their advantages include a low-temperature process, ultralow leakage current^3^, scalability, stability, endurance, low mask count, compatibility with complementary metal-oxide-semiconductor (CMOS) technology^4^, high thermal tolerance^5^, and threshold voltage (*V*_T_) controllability^6^.

Various new and promising applications have recently been demonstrated based on AOS BEOL FETs (e.g., 2T1C gain-cell memory^4^, monolithic three-dimensional (3D) 2T-dynamic random access memory^7,8^, W doping-based high-performance memory^9^, high-density and low-power memory using the incorporation into ferroelectric FET structures^10^, and the low standby power normally-off microcontroller^11^). In these applications, electrical properties of AOS FETs are mostly controlled via modulating the concentration of carrier donors, i.e., oxygen vacancy (*V*_O_) or hydrogen species, which are employed during the deposition of AOS active thin films or by combining the depositions of active film and gate insulator (GI) (especially in the case of the self-aligned top-gate coplanar structure^12–18^). However, these methods are not actually suitable for the BEOL process owing to their complexity and high cost. More BEOL-compatible AOS FET technology is required.

On the other hand, in these applications, whether the degree of integration density of AOS FETs is comparable to CMOSFETs determines the performance, power consumption, and cost. In particular, from the viewpoint of power consumption, AOS FET’s advantage of very low leakage current may be diluted unless the integration density is significantly improved. Therefore, an AOS FET technology, which is compatible with the BEOL process and facilitates FET scaling-down, should be developed.

Meanwhile, as the AOS FETs are integrated with the CMOSFETs in the BEOL process and attract more attention as promising devices that create new and various functionalities, the understanding of the effect of process/structure condition on the short-channel effect (SCE) is urgent. Up to now, the device modeling and simulation have rarely demonstrated a comprehensive optimization of *V*_T_, SCE, subthreshold swing (SS), and field-effect mobility (µ_FE_) of short-channel AOS FETs, particularly in terms of the effect of process/structure condition on SCE.

This study demonstrates the device modeling of submicron amorphous InGaZnO (a-IGZO) FETs, based on experimentally extracted parameters. Both the subgap density of states (DOS), which is extracted through the photo-response of the current–voltage (*I–V*) characteristics, and the lateral profile of donor doping concentration are considered in the proposed model. In addition, the model parameters are incorporated into the technology computer-aided design (TCAD) simulation framework. Through the device simulation, the effects of oxygen (O) partial pressure and device structure on the device parameters are quantitatively investigated, and the feasibility of comprehensive optimization of the device performance parameters, such as *V*_T_, SCE, SS, and µ_FE_, is proved in the bottom-gate (BG) and double-gate (DG) IGZO FETs fabricated with a two-step O annealing. The range of the channel length (*L*) of used device is *L* = 0.245–20.2 µm. In particular, which role the lateral profile of *V*_O_ and O concentrations can play in suppressing either the *L*-dependency of *V*_T_ or the drain-induced barrier lowering (DIBL) is explained and validated based on the proposed model-based simulation.

## Experimental results

### Two-step oxygen anneal-based fabrication of AOS FETs

**Figure 1:**
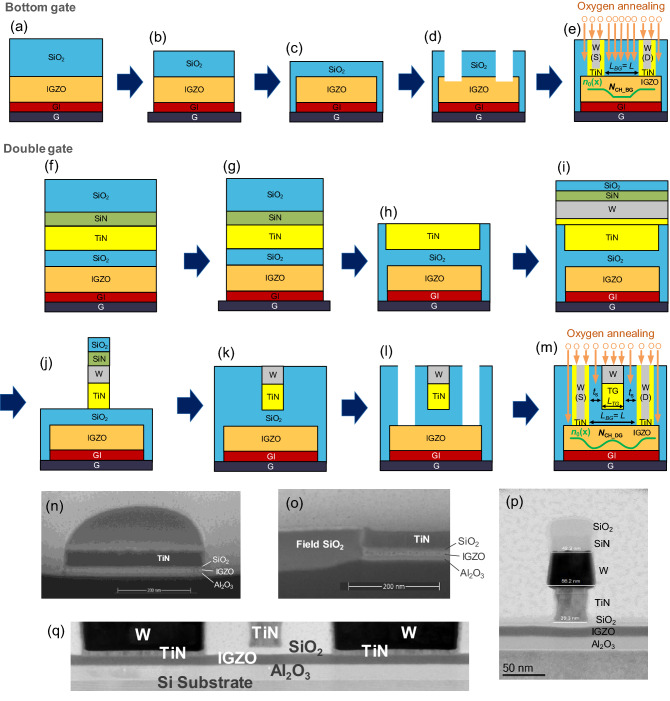
The integration process of the IGZO FET fabrication. Process sequence for (**a**–**e**) BG and (**f**–**m**) DG FET fabrication. SEM images of DG FET (**n**) after active patterning (**g**) and (**o**) after planarization (**h**). TEM images of DG FET (**p**) after gate patterning (**j**) and (**q**) after metallization (**m**).

Figure 1a–e show the process of a bottom-gate (BG) IGZO FET fabrication^19^. First, 5-nm SiCN and 15-nm Al_2_O_3_ films are deposited on a *p*^+^-Si wafer, serving as back gate dielectrics (for BG). Then, a 12-nm-thick a-IGZO film is deposited by using physical vapor deposition (PVD). After that, a 180-nm SiO_2_ hard mask is deposited on top of the IGZO layer. Thus, the full stack is formed (Fig. 1a) and then patterned (Fig. 1b). SiO_2_ is then deposited and planarized (Fig. 1c). Next, the source/drain (S/D) contact trenches are patterned, landing selectively on IGZO (Fig. 1d). Finally, the metallization is implemented by depositing a 6-nm atomic layer deposition (ALD) TiN barrier and ALD/chemical vapor deposition (CVD) W metal contacts followed by a chemical mechanical polishing (CMP). The first oxygen anneal is then performed at 350 °C in an O_2_ atmosphere for 1 h in order to passivate defects generated during the process fabrication (Fig. 1e). The channel length of BG FET (*L*_BG_) is determined by the distance between the source and the drain contact holes. The IGZO active thickness (*t*_act_) and the equivalent oxide thickness of BG dielectric (*t*_BGI_) are 10 nm and 6.5 nm.

On the other hand, Fig. 1f–m show the process of the DG IGZO FET fabrication^20^. First, 15-nm Al_2_O_3_ film is deposited on a *p*^+^-Si wafer, serving as back gate dielectrics (for BG). After a 10-nm-thick a-IGZO film is then PVD deposited, the first oxygen anneal is performed. A top gate dielectric (SiO_2_, 7 nm) and a TiN gate metal are then deposited (for top-gate (TG)) and followed by the deposition of SiCN/SiO_2_ hard mask (Fig. 1f). After the active patterning (Fig. 1g), SiO_2_ gap fill, planarization stopping on TiN, and etch-back are done (Fig. 1h). Then, the rest of the gate stack is deposited (TiN/W/SiCN/SiO_2_) (Fig. 1i), patterned (Fig. 1j), SiO_2_ gap fill, and planarized (Fig. 1k). Next, the S/D contact trenches are opened down to the IGZO layer (Fig. 1l). The contact metals are deposited (TiN-contact barrier/W-metal fill) and planarized. The first oxygen anneal is the same as the BG devices (Fig. 1m). Additional interconnections made of vias and metal lines are then implemented to access small devices. The TG channel length (*L*_TG_) in the DG structure (unlike the *L*_BG_ in the BG FET) is determined by the length of the TG electrode.

The scanning electron microscope (SEM) images after active patterning (Fig. 1g) and after planarization (Fig. 1h) of DG FETs are shown in Fig. 1n,o. In addition, the transmission electron microscopy (TEM) images after gate patterning (Fig. 1j) and after metallization (Fig. 1m) of DG FETs are given in Fig. 1p,q. As seen in Figs. S1–S3 (supplementary information), the wafer map for the critical dimension suggests that the entire fabrication process is reproducible, and a high-yield process is used in this work.

To adjust the donor doping concentration of the channel in IGZO (*N*_CH_), the second oxygen anneal is performed in both DG and BG FETs (Fig. 1e,m). According to the condition of the second oxygen anneal, two types of samples are prepared; (1) 350 °C in an O_2_ atmosphere 1 atm for 2 h (called by the standard (STD) sample) and (2) 350 °C in an O_2_ atmosphere 1 atm for 2 h + 350 °C in an O_2_ atmosphere 20 atm for 2 h (called by the high pressure (HP) sample).

In the BG structure, the S/D metal acts as a diffusion barrier for oxygen during the oxygen annealing process (Fig. 1e). Meanwhile, in the DG structure, both TG metal and the S/D metal act as oxygen diffusion barriers during the second oxygen annealing (Fig. 1m). Therefore, oxygen infiltrates locally through the separation (*t*_s_) between the TG edge and the S/D contact trenches.

As the BG FET goes through the second oxygen annealing process without TG, the IGZO under the S/D metal has a low O concentration (that is, it has a high *V*_O_ concentration). Owing to this concentration difference, *V*_O_ diffusion occurs from the IGZO under the S/D metal toward the center of the channel, and the resulting donor doping concentration [*n*_0_(*x*)] has a laterally non-uniform profile (shown as a green line in Fig. 1e). The lateral profile of *n*_0_(*x*) is a crucial factor in determining the electrical characteristics and the SCE of IGZO FETs, which will be modeled in detail later. Compared to BG FET, the O concentration of the channel is relatively low, and the *N*_CH_ is higher in DG FET (*N*_CH_DG_ > *N*_CH_BG_) because the second oxygen annealing step proceeds in the DG structure while the TG electrode exists. Furthermore, due to O penetrating the *t*_s_, a local high-concentration O region (that is, a locally low *n*_0_ region) is formed on both edges of the *L*_TG_ (shown as the green line in Fig. 1m). This region has a vital role in suppressing the SCE of the submicron IGZO BEOL FET, as will be discussed later.

### IGZO FET DC characterization

**Figure 2:**
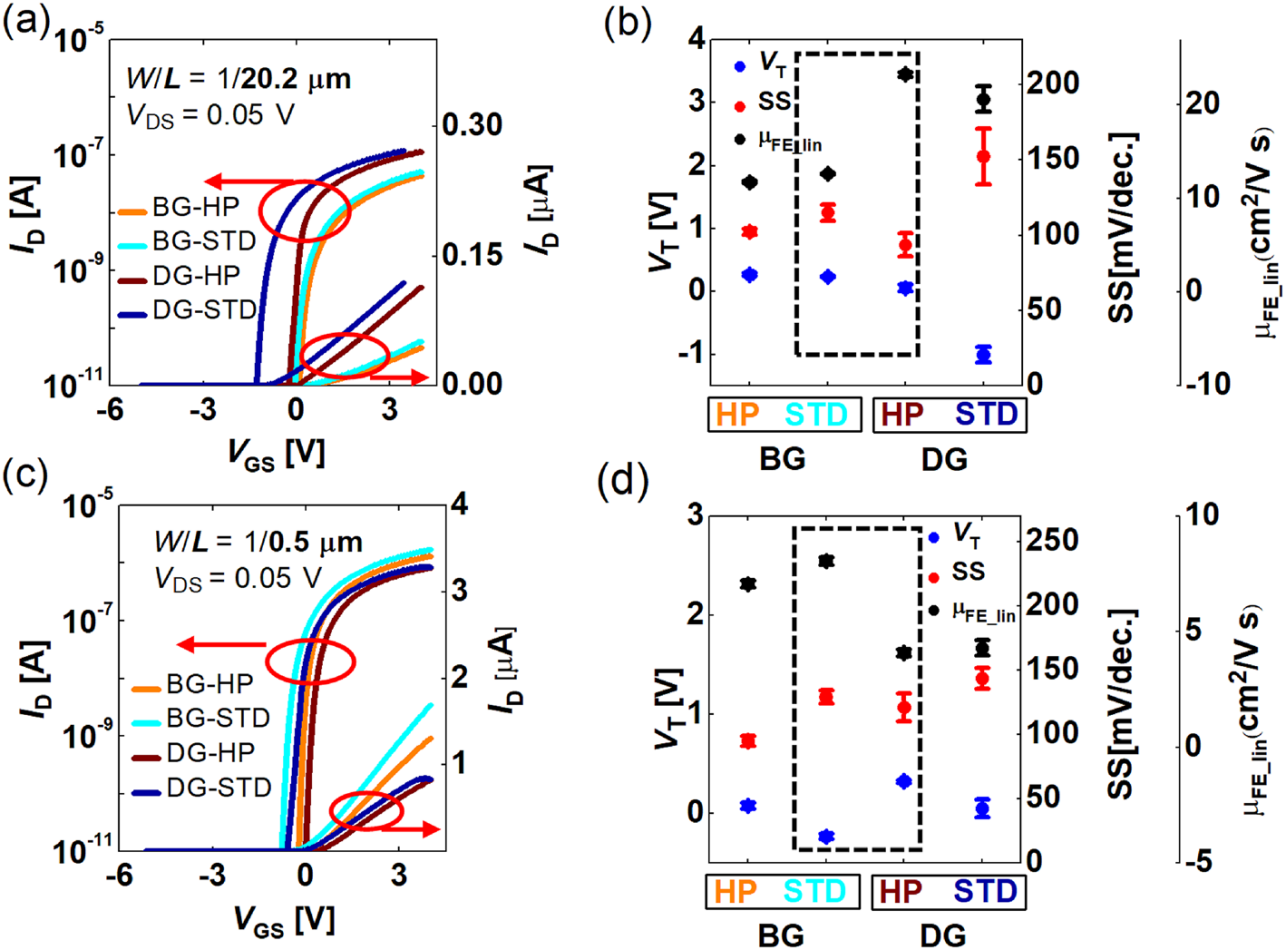
The (**a**) transfer characteristics and (**b**) device parameters (i.e., *V*_T_, SS, and μ_FE_) of long-channel IGZO FETs (*L* = *L*_BG_ = 20.2 μm and *L*_TG_ = 20 μm). The (**c**) transfer characteristics and (**d**) device parameters of short-channel IGZO FETs (*L* = *L*_BG_ = 0.5 μm and *L*_TG_ = 0.3 μm). Error bar was taken from five device samples.

The device sample types are divided into BG and DG according to their structure. They are also divided into STD and HP according to the partial pressure of oxygen during the second O annealing step. The four types of samples are named BG-STD, BG-HP, DG-STD, and DG-HP, respectively. The basic characteristics of the devices were analyzed. Figure 2a,b are the transfer characteristics and device parameters (i.e., *V*_T_, SS, and µ_FE_) for the long-channel devices [channel width (*W*)/channel length (*L*) = 1/20.2 µm] while Fig. 2c,d show the transfer characteristics and device parameters for the short-channel devices (*W*/*L* = 1/0.5 µm). Here µ_FE_lin_ means the µ_FE_ extracted in the linear region (*V*_GS_ = 1 V and *V*_DS_ = 0.05 V). Error bars in Fig. 2b,d were taken from five devices. This supports the reproducibility of device parameters. When measuring the transfer characteristics of the DG FET, the BG voltage (*V*_BG_) was swept, and the TG was floated. *L*_TG_ is 20 µm when *L* = *L*_BG_ = 20.2 µm, and is 0.3 µm when *L* = *L*_BG_ = 0.5 µm, which is due to *t*_s_ = 0.1 µm.

As is demonstrated in Fig. 2b,d, the HP sample has a higher *V*_T_, a smaller SS, and a lower µ_FE_ than the STD sample with the same structure and the same *L*, regardless of device structure or channel length. For the HP sample, because the amount of O inside the IGZO film is larger than that of the STD sample, the *V*_O_ concentration (*n*_Vo_) is lower than the STD sample, and so is the *N*_CH_. Since the *V*_O_ plays the role of electron donor inside the IGZO, the *V*_T_ of the HP sample is relatively high. In addition, since the electron transport in IGZO follows a percolation transport^21^, the mobility increases along with electron concentration. The lower µ_FE_ in the HP sample is due to the lower electron concentration (relatively lower than in the STD sample). The change in the SS due to the amount of O is related to the trap density at the IGZO/GI interface and in the IGZO thin-film bulk; therefore, it can be explained by analyzing the DOS.

### Subgap DOS extraction

**Figure 3:**
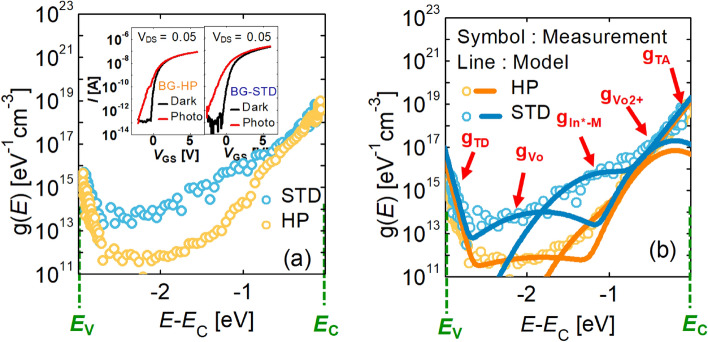
(**a**) The DOS depending on the O content, extracted from the BG IGZO FETs with *W*/*L* = 1/ 20.2 μm. The inset shows the measured photo-response of the *I–V* curve. (**b**) The extracted DOS (symbol) fitted with the proposed DOS model (line).

The DOS was extracted using the photo-response of the *I–V* characteristics of the IGZO FET (inset of Fig. 3a). The detailed procedure for the DOS extraction is described in the supplementary information (including Fig. S4 and S5). Figure 3a demonstrates how the DOS of IGZO, extracted from the long-channel BG FET, depends on the amount of O. The bandgap energy (*E*_g_) of IGZO is 3 eV, and the extracted DOS consists of the following terms, in the order of increasing energy level from the valence band (VB) maximum level (*E*_V_) to the conduction band (CB) minimum level (*E*_C_): *g*_TD_ (valence band tail states), *g*_*V*o_ (neutral *V*_O_ states), *g*_In*−M_ (undercoordinated In states), g_*V*o2+_ (ionized *V*_O_ states), and *g*_TA_ (conduction band tail states). Additionally, Fig. 3b shows that the extracted DOS fits well with the proposed DOS model. The DOS *g*(*E*) model formula is calculated in the following equations, and the extracted DOS model parameters are tabulated in Table S1 in the supplementary information.

1 $$g(E) = g_{A} (E) + g_{D} (E)$$

2 $$g_{A} (E) = g_{TA} (E) + g_{{In^{*} - M}} (E) = N_{TA} \exp \left( { - \frac{{E_{C} - E}}{{kT_{TA} }}} \right) + N_{{In^{*} - M}} \exp \left( { - \left( {\frac{{E_{C} - E_{{In^{*} - M}} - E}}{{kT_{{In^{*} - M}} }}} \right)^{2} } \right)\,$$

3 $$\begin{aligned} g_{D} (E) & = g_{TD} (E) + g_{{V_{o} }} (E) + g_{{V_{o}^{2 + } }} (E) \\ & = N_{TD} \exp \left( { - \frac{{E - E_{V} }}{{kT_{TD} }}} \right) + N_{{V_{o} }} \exp \left( { - \left( {\frac{{E_{V} + E_{Vo} - E}}{{kT_{{V_{o} }} }}} \right)^{2} } \right) \\ & \quad + N_{{V_{o}^{2 + } }} \exp \left( { - \left( {\frac{{E_{C} - E_{{V_{o}^{2 + } }} - E}}{{kT_{{V_{o}^{2 + } }} }}} \right)^{2} } \right) \\ \end{aligned}$$

When the IGZO DOS change by the O annealing conditions was evaluated, the *g*_*V*o_ of the HP sample with high O concentration was confirmed as small compared with the STD sample. The decrease in *V*_O_ explains this well. In addition, *g*_In*−M_, which indicates the density of the states by the combination of the undercoordinated In and metal cation (In^*^–M), was also relatively lower in the HP sample case. Consistently with^22^, this indicates that the In^*^–M probability of bond formation increases as the carrier concentration increases. In addition, *g*_TA_ and g_*V*o2+_ just below the *E*_C_^23–25^ are relatively low in the HP sample case, which is consistent with that the SS of the HP sample is lower than that of the STD sample (Fig. 2b). Noticeably, the O content-dependent DOS in long-channel BG FETs and the device characteristics are well matched; therefore, the extracted DOS is reasonable.

Based on the consistency between DOS and device parameters, it is well understood that under the same O partial pressure condition in a long-channel device, the *V*_T_ of the BG FET is higher than that of the DG FET, and the SS and the µ_FE_ are smaller (Fig. 2a,b). This is because the DG device prevents O from infiltrating (compared with the BG device) into the IGZO during the O annealing process.

On the other hand, the changes by the O content and device structure of *V*_T_, SS, and µ_FE_ become complicated in a short-channel device (Fig. 2c,d). While the O-dependent change tendencies of *V*_T_, SS, and µ_FE_ in the same device structure is the same as the long-channel device, the tendency of their changes between the BG-STD and the DG-HP samples is reversed in the short-channel devices (shown as dotted rectangles in Fig. 2b,d). Undoubtedly, this is because the *V*_T_ of the sample, which experienced the same O annealing condition, varied with *L*.

The understanding of the *L*-dependence of *V*_T_ is eventually central to a robust design of submicron AOS FETs. To understand either the SCE of IGZO FETs or the effect of O content and structure on the SCE, appropriate device modeling and simulation are necessary and should be supported for a robust design of a submicron IGZO FET immune to the SCE.

## Modeling and simulation

### Device model and parameter extraction

**Figure 4:**
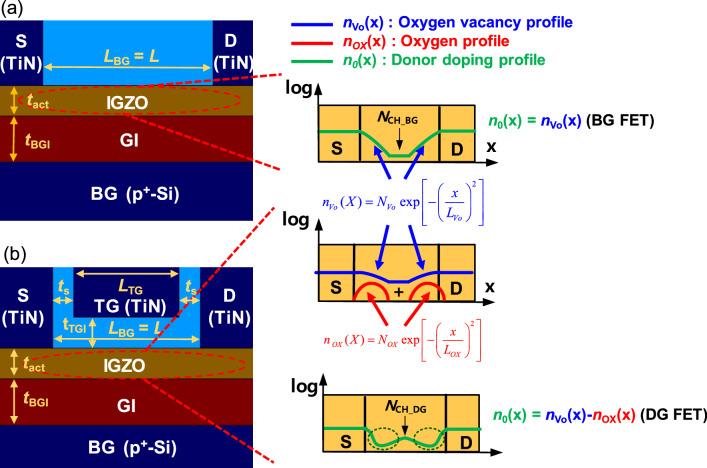
Models for *n*_Vo_(*x*) and *n*_0_(*x*) considering the device structure and process conditions, which are implemented by the TCAD device simulator for the (**a**) BG FET and the (**b**) DG FET structure.

To obtain the robust framework for material-process-device co-design of submicron IGZO FETs, we performed device modeling and incorporated it into a TCAD simulation. First, as is shown in Fig. 4, the BG FET and DG FET device structures were implemented with the TCAD, and the *n*_Vo_(*x*) and *n*_OX_(*x*) profiles were generated along with the O annealing process conditions and modeled by the equations in Fig. 4. Here *n*_OX_ is the concentration of oxygen which is additionally employed during the second oxygen anneal in DG FETs.

In our modeling, the *n*_Vo_(*x*) and *n*_OX_(*x*) were assumed to be Gaussian profiles through the previous consideration of the two-step O annealing process and the STD/HP conditions. The *L*_Vo_ and *L*_OX_ mean characteristic lengths, which are associated with the respective diffusivity of *V*_O_ and O during the oxygen annealing, describing the *n*_Vo_(*x*) and *n*_OX_(*x*). Final donor doping profile [*n*_0_(*x*)] of the BG FET is determined by the combination of the *n*_Vo_(*x*) near the S/D extension and the *N*_CH_BG_ in the center of a channel. In contrast, the final *n*_0_(*x*) of the DG FET is determined as *n*_Vo_(*x*)−*n*_OX_(*x*) near the S/D extension and the *N*_CH_DG_ in the channel part. Then, the *N*_OX_ was determined as *N*_OX_ = *N*_CH_BG_ − *N*_CH_DG_ because the additional oxygen would compensate the donor concentration.

The detailed procedure for extracting the model parameters is described in Fig. S6 in the supplementary information. The process is outlined as follows. First, after inputting the material parameters and the device structural parameters into the TCAD, we designated the experimentally extracted DOS model into the IGZO active layer. Based on the DOS parameter, the electron effective mass (*m*_n_*) and the CB effective DOS (*N*_C_) were determined through the formulas below^26^.

4 $$m_{n}^{*} = \left( {\frac{{N_{TA} }}{{4.9 \times 10^{21} \sqrt {kT_{TA} } }}} \right)^{\frac{2}{3}} \times m_{0}$$

5 $$N_{C} = 4.9 \times 10^{21} \left( {\frac{{m_{n}^{*} }}{{0.34m_{0} }}} \right)^{\frac{3}{2}}$$

Then, the *N*_CH_ and the charge density in GI (*Q*_ox_) were fitted by comparing the simulated *V*_T_ and flat band voltage (*V*_FB_) and the measured ones. After that, the CB mobility (µ_band_) was adjusted by the numerical iteration until the simulated *∂I*_D_/*∂V*_GS_ and measured *∂I*_D_/*∂V*_GS_ were in good agreement at various *V*_DS_’s. In these fitting processes, the numerical iteration was allowed until the average error between the simulated and measured values fell within a specific error rate (ER). The ER can be selected considering the trade-off between the precision of the model parameter and the computing burden of simulation. An ER = 10% was used in this study.

**Figure 5:**
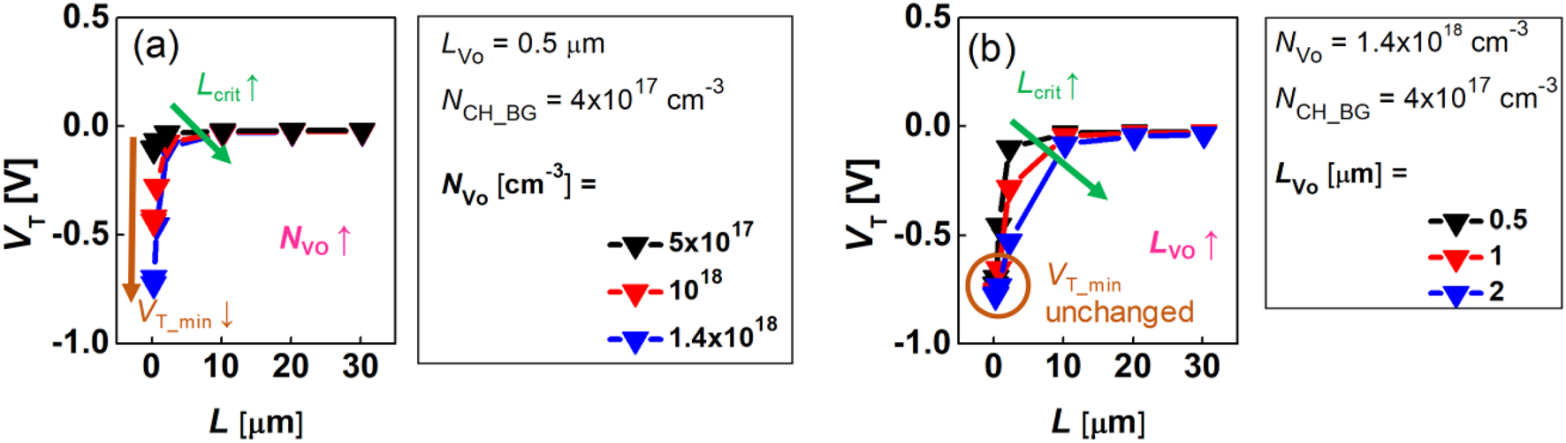
The TCAD simulation results for the *L*-dependent *V*_T_ in BG FETs with the variations of (**a**) *N*_Vo_ and (**b**) *L*_Vo_.

Next, the parameters that defined the lateral profile of the carrier doping concentration (e.g., *N*_Vo_, *L*_Vo_, *N*_OX_, and *L*_OX_) were decided. These parameters were extracted using the point that they affected the *V*_T_ roll-off characteristic, i.e., the *L*-dependency of *V*_T_, of the short-channel FET. Since the *n*_0_(*x*) of the BG FET was determined by the *n*_Vo_(*x*), we performed the simulation study (Fig. 5) to understand the effect of *N*_Vo_ and *L*_Vo_ changes on the *V*_T_ roll-off characteristic.

As is shown in Fig. 5, the *V*_T_ roll-off occurs as *L* becomes shorter in the BG FET. Here, the channel length at which the *V*_T_ roll-off begins is defined as *L* = *L*_crit_, and the *V*_T_ at the minimum *L* (*L* = 0.245 µm) is defined as *V*_T_min_. As demonstrated in Fig. 5a, as the *N*_Vo_ becomes higher, the *L*_crit_ becomes longer, and the *V*_T_min_ becomes lower simultaneously. It is also shown in Fig. 5b that as the *L*_Vo_ becomes longer, the *L*_crit_ becomes more extended, but *V*_T_min_ is independent of the *L*_Vo_. Therefore, in the BG FET, the *N*_Vo_ can be extracted through a fitting with the measured *V*_T_min_ under a fixed *N*_CH_BG_ condition, and then the *L*_Vo_ can be extracted through the second fitting with the measured *L*_crit_ at a fixed *N*_Vo_.

**Figure 6:**
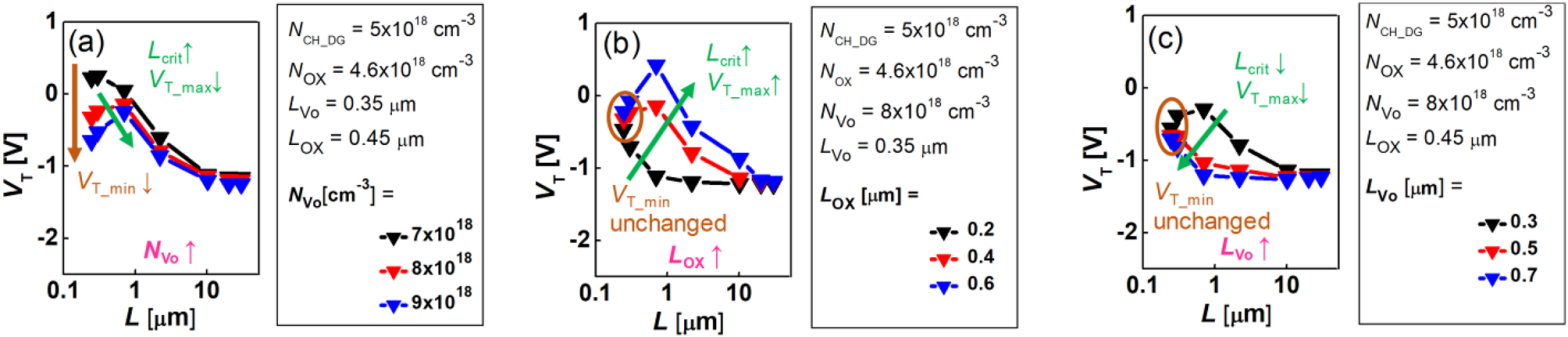
The TCAD simulation results for the *L*-dependent *V*_T_ in DG FETs by splitting the (**a**) *N*_Vo_, (**b**) *L*_OX_, and (**c**) *L*_Vo_.

In the DG FET, the changes either in *N*_Vo_ and *L*_Vo_ or in *N*_OX_ and *L*_OX_ affect the *V*_T_ roll-off characteristic since the *n*_0_(*x*) is determined by the combination of the *n*_Vo_(*x*) and the *n*_OX_(*x*). Therefore, in the DG FET, a more complex *L*-dependency of the *V*_T_ appears compared with the BG FET as seen in Fig. 6. More interestingly, the reverse SCE appears in the medium *L* range where the *V*_T_ increases as the *L* decreases. Here, when the maximum value of the *L*-dependent *V*_T_ is defined by *V*_T_max_, *L*_crit_ can be re-defined particularly in DG FET as the *L* where the *L*-dependency of *V*_T_ interchanges between the increase and decrease in *V*_T_(*L*). Then, as the *N*_Vo_ increases, *V*_T_min_ decreases, *L*_crit_ increases, and *V*_T_max_ decreases (Fig. 6a). In addition, as *L*_OX_ becomes longer, both *L*_crit_ and *V*_T_max_ increase, but the *V*_T_min_ is independent of the *L*_OX_ (Fig. 6b). Moreover, as *L*_Vo_ lengthens, both *L*_crit_ and *V*_T_max_ decrease, but the *V*_T_min_ is immune to the variation of *L*_Vo_ (Fig. 6c).

Therefore, in the DG FET, the parameters, such as *N*_Vo_, *L*_Vo_, *N*_OX_, *L*_OX_, and *N*_CH_DG_, can be extracted through the relationship of *N*_OX_ = *N*_CH_BG_ − *N*_CH_DG_ and a fitting between the measured *V*_T_min,_ *L*_crit_, and *V*_T_max_, and simulated values under the given *N*_CH_BG_ condition.

The extracted model parameters are summarized in Table S1 (supplementary information).

### TCAD simulation

**Figure 7:**
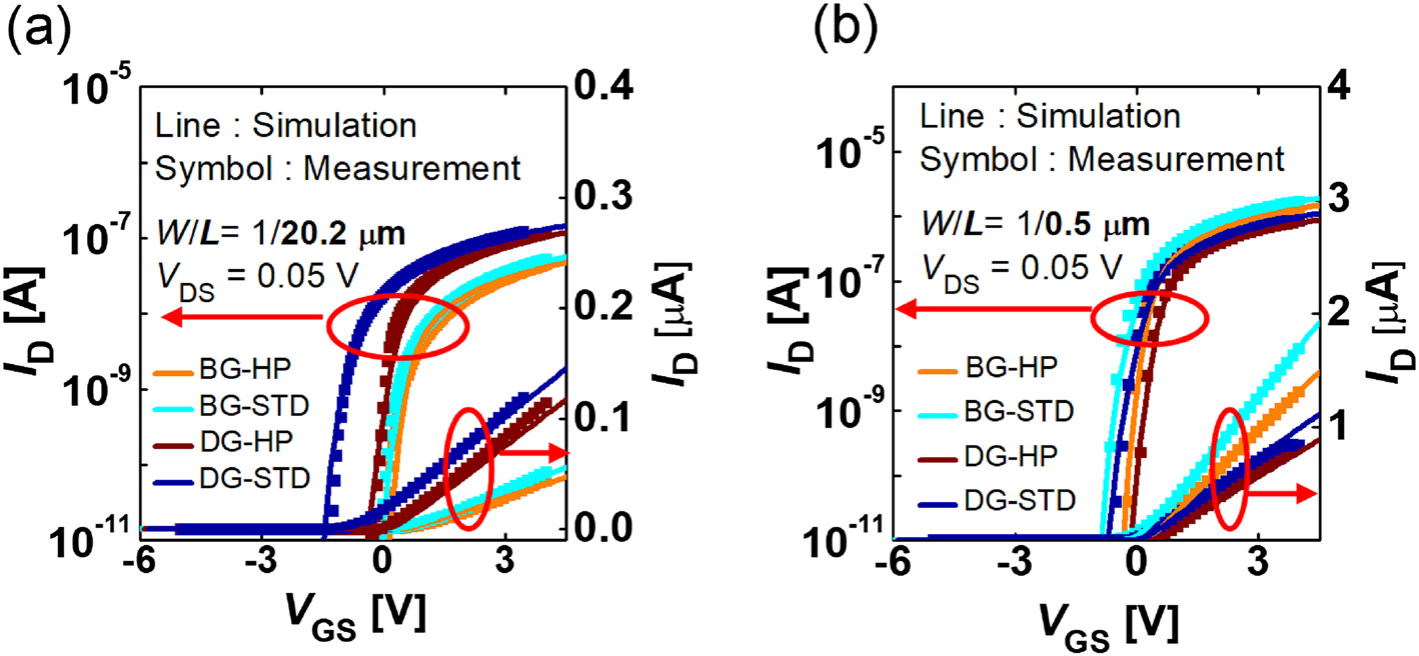
The TCAD simulation (line) vs. the measurement data (symbol). (**a**) *I–V* curve at *W*/*L* = 1/20.2 μm, and (**b**) *I–V* curve at *W*/*L* = 1/0.5 μm.

The TCAD framework was setup for each process condition (HP/STD) and structure (BG/DG) by using the parameter set summarized in Table S1. The simulated and measured *I–V* characteristics are compared with each other (Fig. 7a,b). In the four types of samples (BG-STD, BG-HP, DG-STD, and DG-HP), all the TCAD simulation results reproduce well the measured *I–V* curves and parameters, i.e., *V*_T_, SS, and µ_FE_, for both the long-channel (Fig. 7a) and short-channel FETs (Fig. 7b).

Considering the discrepancies between the measured and simulated results, the ER range is 1.16–13.1%. The main cause of this difference is expected to be due to the bias-dependent mobility of the AOS FETs, suggesting that further studies are needed. In Fig. 7a (*L *= 20.2 μm), the ER for BG-HP, BG-STD, DG-HP, and DG-STD is 7.35, 5.57, 13.1, and 3.82%, respectively. Meanwhile, in Fig. 7b (*L* = 0.5 μm), each ER for BG-HP, BG-STD, DG-HP, and DG-STD is 1.16, 3.67, 8.65, and 9.48%.

These values are consistent with the critical ER (< 10%) used in extracting model parameters. Therefore, the proposed model and model parameter extraction are reasonable and accurate enough to expect the electrical characteristics of AOS FETs.

Noticeably, using the *L* vs. *V*_T_ relationship in Figs. 5 and 6, we can extract the Gaussian doping parameters for each structure and process condition (Fig. 4) as a unique set. This means that the O and *V*_O_ diffusion effects are reflected well along with every *L*, process condition, and structure (including the minimum *L* of 245 nm). As seen in Table S1, in the long *L*, the *N*_CH_ was higher in the order of DG-STD, DG-HP, BG-STD, and BG-HP. In the second O annealing step of the BG structure, since there is no O diffusion barrier, *N*_CH_DG_ is higher than *N*_CH_BG_. In the same structure, the *N*_CH_ of the STD device is higher than that of the HP device.

## Discussion

### Short-channel effects

Understanding why the *L*-dependency of *V*_T_ in the BG FET changes along the variations of *L*_Vo_ and *N*_Vo_ (Fig. 5) is central to the design of the doping profile of the submicron AOS FET. Similarly, understanding why the *L*-dependency of *V*_T_ of the DG FET changes along with the variations of *L*_Vo_, *N*_Vo_, and *L*_OX_ (Fig. 6) is central to the design of the immunity to SCE in the submicron AOS FET.

First, for the BG FET, the *n*_0_(*x*) is composed of *N*_Vo_ (S/D region), $n_{{V_{O} }} (x) = N_{{V_{O} }} \times e^{{ - \left( {\frac{x}{{L{\kern 1pt}_{Vo} }}} \right)^{2} }}$ (S/D extension area), and *N*_CH_BG_ (the center of the channel), as illustrated in Fig. 4a. At this time, the *N*_Vo_ is determined by the amount of the *V*_O_ employed in the IGZO film, which is controlled by the amount of the O inflow and the temperature during the IGZO deposition. The diffusion length of *V*_O_s (*L*_Vo_) is also determined by either the lateral diffusivity of *V*_O_ or the thermal budget during the process. In the same process conditions, when the *L* becomes shorter and comparable to the scale of the *L*_Vo_, then the *n*_Vo_(*x*) profiles of both the source and drain merge in the center of the channel to effectively increase the *N*_CH_; therefore, the *V*_T_ starts to decrease. That is, when the *L* = *L*_crit_, the *N*_CH_BG_ increases compared with the long-length device, and the *V*_T_ roll-off phenomenon begins to occur. Accordingly, as the *L*_Vo_ lengthens and the *N*_Vo_ becomes higher, the longer *L*_crit_ becomes (the SCE becomes severe) because the *n*_Vo_(*x*) starts to intersect at the channel center at the longer *L* (Fig. 5a). At the minimum *L* (*L* = 0.245 µm), the channel length is too short, and the donor doping concentration in the center of the channel approaches *N*_Vo_, i.e., the peak value of the Gaussian donor doping *n*_Vo_(*x*), independently of the *L*_Vo_. Therefore, the *V*_T_min_ is only affected by *N*_Vo_ (Fig. 5b).

On the other hand, in the DG FET (Fig. 6), if the locally high O concentration area (formed during the second O annealing step) is described as the Gaussian acceptor profile $n_{OX} (x) = N_{OX} \times e^{{ - \left( {\frac{x}{{L_{{{\kern 1pt} OX}} }}} \right)^{2} }}$, then the *n*_0_(*x*) consists of the *N*_Vo_ in the S/D area, the *n*_Vo_(*x*) − *n*_OX_(*x*) in the S/D extension area, and the *N*_CH_DG_ in the center of the channel. At this time, the O diffusion length *L*_OX_ is determined by either the lateral diffusivity of O or the thermal budget during the process. Since the *n*_OX_(*x*) region is relatively closer to the channel center than the *n*_Vo_(*x*) region, when *L* is shortened, the locally higher *n*_OX_(*x*) [locally lower *n*_0_(*x*)] regions are merged first, and then *V*_T_ increases. That is, as shown in Fig. 6, a *V*_T_ roll-up (the reverse SCE) is observed (*V*_T_max_ is observed).

Furthermore, as *L* becomes further shorter, the merge in the *n*_Vo_(*x*) section is overlapped. Then, a *V*_T_ roll-off section, where *V*_T_ decreases again, is observed. Meanwhile, as *L*_Vo_ increases, the *N*_CH_DG_ also increases and *V*_T_max_ decreases. In this case, as *L*_Vo_ increases, *L*_crit_ shortens since the influence of the *n*_OX_(*x*) also decreases (Fig. 6a). In addition, as *L*_OX_ lengthens, *V*_T_max_ increases because the *N*_CH_DG_ decreases. However, *L*_crit_ lengthens because the *N*_CH_DG_, which has already been lowered, makes the *n*_Vo_(*x*) overlap followed by the decrease in *V*_T_ occur at a longer *L* (Fig. 6b). Finally, at the minimum *L* (*L* = 0.245 µm), the *N*_CH_DG_ (also *V*_T_min_) is determined by the combination of the *N*_Vo_ and *N*_OX_ regardless of the *L*_OX_ and *L*_Vo_ because the channel length is too short (Fig. 6b,c). Thus, when the *N*_OX_ is fixed, *L*_crit_ shortens and *V*_T_max_ decreases as the *L*_Vo_ lengthens (Fig. 6c).

**Figure 8:**
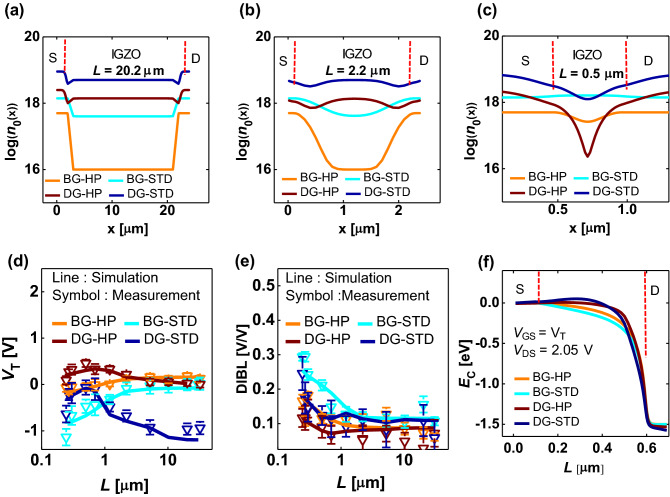
The TCAD-simulated *n*_0_(*x*) profiles in the four types of samples along the structures and processes with varying channel length. (**a**) *L* = 20.2 μm, (**b**) *L* = 2.2 μm, and (**c**) *L* = 0.5 μm. The TCAD simulated (line) vs. the measured (symbol) *L*-dependent (**d**) *V*_T_ and (**e**) DIBL. Error bar was taken from six device samples. (**f**) The energy band obtained through the TCAD simulation for the BG-HP, BG-STD, DG-HP, and DG-STD devices at *W/L* = 0.5/1 µm.

Hence it needs to be explained why, in a short-channel case, the *V*_T_ of the DG FET increases overall and the µ_FE_ decreases overall compared with the BG FET (the dotted boxes in Fig. 2b,d). As explained above, the *N*_CH_DG_ is higher than *N*_CH_BG_ in the long *L*. Therefore, the DG device has a lower *V*_T_ and a higher µ_FE_ when compared with the BG device. However, in a short-channel case, the *N*_CH_DG_ is lower than that of the *N*_CH_BG_ due to the merging phenomenon of the *n*_OX_(*x*), so that the DG device has a higher *V*_T_ and a lower µ_FE_ than the BG device (Fig. 8a–c). Furthermore, at the same process condition, the SS of the DG device is higher in the short channel (*L* = 0.5 µm) than that of the BG device, whereas the SS in the long channel is immune to the device structure and process. This is because in the DG device with a short *L*, there is a big difference between the resistance of the channel and that of the S/D extension (Fig. 8a–c), so the lateral electric field by the drain voltage becomes more focused on the channel center, and the vertical electric field by the gate voltage acts relatively weakly; therefore, this weakens the gate controllability.

The symbols in Fig. 8d,e show the *L*-dependent *V*_T_ and DIBL characteristics measured in the IGZO FET. Error bars were taken from six devices. These two characteristics are the performance index representing the SCE. In addition, the lines in Fig. 8d,e show the TCAD simulation results (using the model parameters in Table S1) reproduce well the *L*-dependent *V*_T_ and DIBL characteristics over a wide range of the *L*. Based on these results, the model parameter extraction process and the parameter values confirm that the O and *V*_O_ diffusion effects are reflected well over a wide range of the *L*. This is also reasonable for the variations of process condition and device structure, both qualitatively and quantitatively.

In Fig. 8d, when the *V*_T_ roll-off occurs in the BG FET, the HP device is more immune to the SCE than in the STD device (that is, the *L*_crit_ is longer). This is because the HP device has a relatively lower *N*_CH_ and *N*_Vo_ than the STD device. Here, it is worthwhile to note that the *L*_Vo_ of HP device is × 1/5 to × 1/3 shorter than that of STD device (Table S1). It indicates that the more O-poor the IGZO active layer is, the more active the lateral diffusion of the *V*_O_ and the longer the diffusion length of the *V*_O_ will be. Our finding is consistent with the results of studies that indicate that the diffusion length of the *V*_O_ increases when the *N*_Vo_ is high^27^.

Meanwhile, in the DG FET (as was already shown in Fig. 6), the *V*_T_ roll-up (the reverse SCE) is observed along with a decreasing *L*, and the DG-HP device suppresses the SCE more effectively than the DG-STD device. As shown in *N*_OX_ and *L*_OX_ in Table S1, the *n*_OX_(*x*) spreads more broadly in the DG-HP device, which lowers *N*_OX_ and lengthens *L*_OX_, when compared with the DG-STD device. This broadening further reduces the *N*_CH_DG_ (Fig. 8c), and both the *V*_T_ roll-off and roll-up are consequently suppressed by weakening the lateral-merge effect of the *n*_Vo_ and the *n*_OX_. Therefore, if the DG-HP device is optimized further, it is expected to effectively suppress the SCE while satisfying a positive *V*_T_, which is an essential requirement from the circuit aspect.

### Device design perspective

The following points should be noted: the spread of the *n*_Vo_ in the first O annealing step increases as it becomes more O-poor (the *n*_Vo_ spread in the STD device is larger than that in the HP device), and the spread of the *n*_OX_ in the second O annealing step increases as it becomes more O-rich (broader *n*_OX_ in the DG-HP device rather than in the DG-STD device). Therefore, if the O pressure during the second annealing step and the *t*_s_ are further optimized in the two-step O annealing process, we can expect that the *V*_T_ will become more immune to the scale-down of *L*.

The TCAD-simulated energy band is shown in Fig. 8f. The DIBL is small in the following device order: DG-HP, BG-HP, DG-STD, and BG-STD, which is consistent with Fig. 8e. The overall tendency shows that the HP device has a smaller DIBL than the STD device, and the DG structure has a smaller DIBL than the BG structure. The HP structure is more robust to DIBL than the STD structure for the following two reasons; first, since the HP structure has a low *N*_CH_ and the Fermi energy level (*E*_F_) is lower than that of the STD structure, a high energy barrier is formed between the S/D and the channel. Second, the *L*_Vo_ of the HP device is shorter than that of the STD device. If the *L*_Vo_ is long, the channel area where the *E*_F_ rises gets longer, and the potential barrier lowers between the source and the channel, which makes it more vulnerable to the DIBL.

In the DG devices, the voltage drop by the *V*_DS_ is large because of the existence of a *n*_OX_ region with large resistance. Thus, the DIBL is suppressed in DG FET. This effect is further enhanced in the HP process (the resistance of the *n*_OX_ region becomes larger). The role of this *n*_OX_ region is very reminiscent of the role of halo (or pocket) implantation to block SCE in submicron CMOSFETs. The DG-HP device is the most advantageous from the DIBL point of view (similar to the *V*_T_ roll-off), and the DIBL is expected to be suppressed more effectively through further optimization.

To compare the performance of devices utilized in this study with previous studies, we compared the main device parameters with those of references, as summarized in Table 1. It should be noted that, since the operating voltage, device structure, and size are different for each device, it is necessary to define another new index for a fair comparison. In the case of mobility, for example, since either the voltage condition from which the mobility is extracted or the channel length is different, it is difficult to evaluate as the performance indicator of AOS. Therefore, we defined α and β as new indices. α is defined by $\alpha = \frac{{I_{D} \times L_{\min } }}{{W \times (V_{GS} - V_{T} ) \times V_{DS} }}$ and means the operating current normalized by the device size, gate overdrive voltage, and drain voltage. β is also defined by $\beta = \frac{\alpha }{{C_{ox} }}$, means the current further normalized by *C*_ox_, and has the same unit as the mobility. That is, if β is used, fair comparison is possible even if the operating voltage, gate capacitive coupling, and device size are different. In the case of our device, assessed by β, it is confirmed that the performance is excellent compared to the devices of the previous studies. Furthermore, in the viewpoint of *L*_min_, it is found that our device shows good scalability except for the 3D device structure such as FinFET. From the SCE point of view, it can be seen that the previous studies rarely reported on *V*_T_ roll-off or DIBL. Noticeably, our study is a rare case that reports both DIBL and *V*_T_ roll-off, and it is confirmed that our device shows very good SCE properties despite not having a 3D structure.

**Table 1:** Summary of the performance indices compared with previous studies.

Index Ref.	Device structure	*A* [μA/V^2^]	*B* [cm^2^/V⋅s]	*V*_T_ [V]	μ [cm^2^/V⋅s]	SS [mV/dec]	*L*_min_ [nm]	DIBL (*L*_min_) [V/V]	*V*_T_ (*L*_max_) – *V*_T_ (*L*_min_) [V]
This work	DG/BG	2.56	7.84	0.3	8	94	245	0.1	− 0.1 (DG) 0.19 (BG)
^3^	FinFET	4.31	7.5	0.9	10.3	100	72	–	–
^5^	FinFET	2.33	4.05	1.44	10.2	79	25	–	–
^6^	FinFET	2.05	3.57	0.9	10.5	87	21	0.1	–
^7^	TG	0.11	0.23	− 0.8	–	–	45	–	0.1
^10^	GAA	1.4	1.18	− 1.1	10	100	20,000	–	–
^12^	TG	0.27	8.04	0.55	12.1	100	6500	–	–
^13^	TG	–	–	0.9	8.4	110	6000	–	–
^15^	TG	0.15	4.34	0.9	10.5	180	6000	–	–
^17^	TG	–	–	0.9	6	160	15,000	–	–
^18^	TG	0.45	13.03	0.1	7.5	–	6000	–	–
^19^	BG	–	–	− 0.4	–	175	245	–	–
^23^	BG	–	–	0.5	5.1	105	15,000	–	–
^24^	BG	–	–	0.2	9	274	10,000	–	–

**Figure 9:**
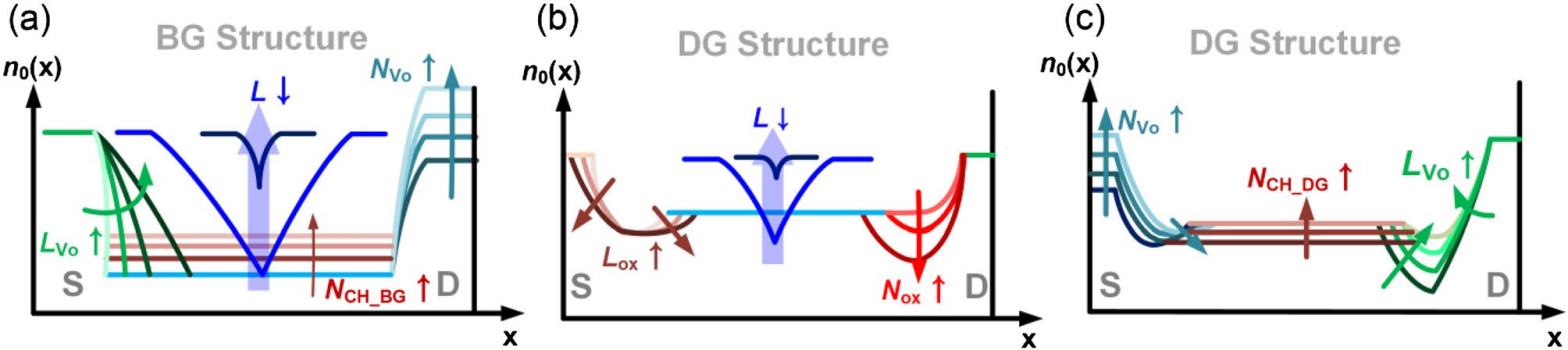
(**a**) Change of *n*_0_(*x*) by the variations of *L*_Vo_, *N*_Vo_, *N*_CH_BG_, and *L* in the BG structure. (**b**) Change of *n*_0_(*x*) by the variations of *L*, *L*_OX_, and *N*_OX_ in the DG structure. (**c**) Change of *n*_0_(*x*) by the variations of *N*_Vo_, *N*_CH_DG_, and *L*_Vo_ in the DG structure.

A method that can optimize *V*_T_, SS, μ_FE_ and SCE in of the submicron AOS FET is vital, and the key is to control the *n*_0_(*x*) with a combination of *V*_O_ and O. Figure 9 illustrates the change of *n*_0_(*x*) by the variations of *L*_Vo_, *N*_Vo_, *N*_CH_BG_, and *L*. If we perform the co-optimization of *t*_s_, *N*_Vo_, *L*_Vo_, *N*_OX_, and *L*_OX_ by using both a two-step O annealing and DG structure after we make the IGZO active film sufficiently O-poor and fully raise the CB mobility at the beginning of the process, we will be able to implement AOS BEOL FETs that comprehensively satisfy the *V*_T_, SS, μ_FE_, and SCE parameters that match the performance and specification of the application-dependent circuits.

## Conclusion

The DOS-based device model and TCAD simulation framework were proposed with emphasis on the control of *n*_0_(*x*), and were demonstrated in the BG/DG IGZO FETs with *L* = 0.245–20.2 µm. The validity of the device model and its parameters were proved through a TCAD simulation reflecting the extracted model parameters and the IGZO DOS. Based on a two-step O annealing, the effect of O partial pressure on not only the device performance parameters, such as *V*_T_, SCE, SS, and µ_FE_, but also DOS was elucidated. The simulation results explained successfully the measured *V*_T_ roll-off and DIBL characteristics. The results of an analysis based on the proposed model and the extracted parameters indicate that the SCE of submicron AOS FETs is effectively suppressed when the local high O-concentration region (formed by applying the two-step O annealing to the DG FET) is used. We also found that *L*_Vo_ becomes longer as the device becomes O-poor, and *L*_OX_ becomes longer as the device becomes O-rich.

Our results show that the co-optimization of *t*_s_, *N*_Vo_, *L*_Vo_, *N*_OX_, and *L*_OX_ is vital to the immunity to SCE in AOS FETs. Proposed model and simulation framework are potentially useful to a comprehensive optimization of the device performance parameters, i.e., *V*_T_, SCE, SS, and µ_FE_, for the submicron AOS BEOL FET technology.

## Methods

### IGZO FET characterization

In this study, to analyze the *I*_D_*−V*_GS_ characteristics of the IGZO FET, HP4156C (Keithley, Santa Rosa, CA, USA) was used, and the measurements were taken in a dark state at room temperature. The measurement conditions were as follows; the gate-to-source voltage (*V*_GS_) was swept from 4 to − 6 V in − 0.05 V steps, and the drain-to-source voltage (*V*_DS_) was fixed at 0.05 V. The *V*_T_ was extracted by the *V*_GS_ at *I*_D_/(*W/L*) = 10^−8^ A, and the SS was extracted by *I*_D_/(*W/L*) = 10^−10^–10^−9^ A. In addition, the µ_FE_lin_ was calculated at *V*_GS_−*V*_T_ = 3 V by using the following equation.

$$\mu_{FE\_lin} = \frac{{dI_{D} }}{{dV_{GS} }} \times \frac{L}{{W \times V_{DS} \times C_{ox} }}$$

The DIBL was calculated from the difference between the *V*_T_ at *V*_DS_ = 0.05 V and the *V*_T_ at *V*_DS_ = 1.05 V.

### Error evaluation

In the case of measured data, the mean value and error bar were calculated by using the root mean square (RMS) value as follows (*x* = measurement value, *m* = mean, *n* = number of data):

$${\text{Error}}\;{\text{bar }} = \sqrt {\frac{{\sum\nolimits_{{}}^{n} {\left( {x - m} \right)^{2} } }}{n}} \quad {\text{and}}\quad m = \frac{{\sum\nolimits_{{}}^{n} {\left| x \right|} }}{n}.$$

These mean and error bar were used in Fig. 2b,d, and in Fig. 8d,e.

In addition, for comparing the measured and simulated data, the error rate (ER) was calculated as follows (*y* = simulation data, *m*_abs_ = average of absolute values of measured data).

$${\text{RMS }} = \sqrt {\frac{{\sum\nolimits_{{}}^{n} {\left( {y - x} \right)^{2} } }}{n}} ,\quad m_{{{\text{abs}}}} = \frac{{\sum\nolimits_{{}}^{n} {\left| x \right|} }}{n},\quad {\text{ and}}\quad {\text{ER }} = \frac{RMS}{{m_{abs} }} \times 100\;(\% )$$

This ER was used in Fig. 7.

## Supplementary Information

Supplementary Information.

## Data Availability

The datasets used or analyzed during the current study are available from the corresponding author on reasonable request.
