# Supplementary material for: Device modeling of two-steps oxygen anneal-based submicron InGaZnO back-end-of-line field-effect transistor enabling short-channel effects suppression
Source: Sci Rep. 2022 Nov 12;12:19380. doi: 10.1038/s41598-022-23951-x (PMC9653482; doi:10.1038/s41598-022-23951-x)
Supplement: Supplementary file 1 — Supplementary Information. [file 41598_2022_23951_MOESM1_ESM.doc]

**Supplementary information**

**Device modeling of two-steps oxygen anneal-based submicron InGaZnO back-end-of-line field-effect transistor enabling short-channel effects suppression**

# Donguk Kim‡,1, Je-Hyuk Kim‡,1, Woo Sik Choi1, Tae Jun Yang1, Jun Tae Jang1, Attilio Belmonte2, Nouredine Rassoul2, Subhali Subhechha2, Romain Delhougne2, Gouri Sankar Kar2, Wonsok Lee3, Min Hee Cho3, Daewon Ha3, and Dae Hwan Kim1,*

1School of Electrical Engineering, Kookmin University, Seoul, 02707, Republic of Korea.

2imec, Kapeldreef 75, B-3001 Leuven, Belgium.

3Advanced Device Research Lab, Semiconductor R&D Center, Samsung Electronics Company, Hwaseong-si, Gyeonggi-do, 18448, Republic of Korea.

*drlife@kookmin.ac.kr

‡ These authors contributed equally.

**Process reproducibility and yield.** To confirm the reproducibility and uniformity of the fabrication process, we checked the scanning electron microscope (SEM) and/or transmission electron microscopy (TEM) images for the critical dimensions (CDs) of either the patterned width or deposited thickness whenever the critical process steps were carried out. As shown in Figs. S1−S3, our results suggest that the entire fabrication process is reproducible, and a high-yield process is used in this work.

**Figure S1.** SEM images and the CD wafer map for BG IGZO FETs. (a) After active patterning [Fig. 1(b)]. (b) After metallization [Fig. 1(e)].

**Figure S2.** SEM images and the CD wafer map for DG IGZO FETs. (a) After active patterning [Fig. 1(g)]. (b) After planarization and etch-back [Fig. 1(h)].

**Figure S3.** SEM images and the CD wafer map for DG IGZO FETs. (a) After gate patterning [Fig. 1(j)]. (b) After the deposition of interlayer dielectric (ILD). (c) After metallization [Fig. 1(m)].

**DOS extraction.** The DOS of IGZO film was extracted using the photo-response of the *I-V* characteristics of the IGZO FETs. Fig. S4(a) and (b) show a photograph and schematic illustration for measuring the photo-response of the *I-V* characteristics. The wavelength (λ) of a monochromatic light source is 440 nm, and the incident photons have smaller energy (2.82 eV) than the bandgap energy of the IGZO (3 eV). Based on the BG structure, a light is irradiated between the source and drain so that a photo-excitation occurs entirely in the FET channel. In Fig. S4(c) and (d), the measured dark and photonic *I*D−*V*GS curves at *V*DS=0.05 V are compared with each other.

**
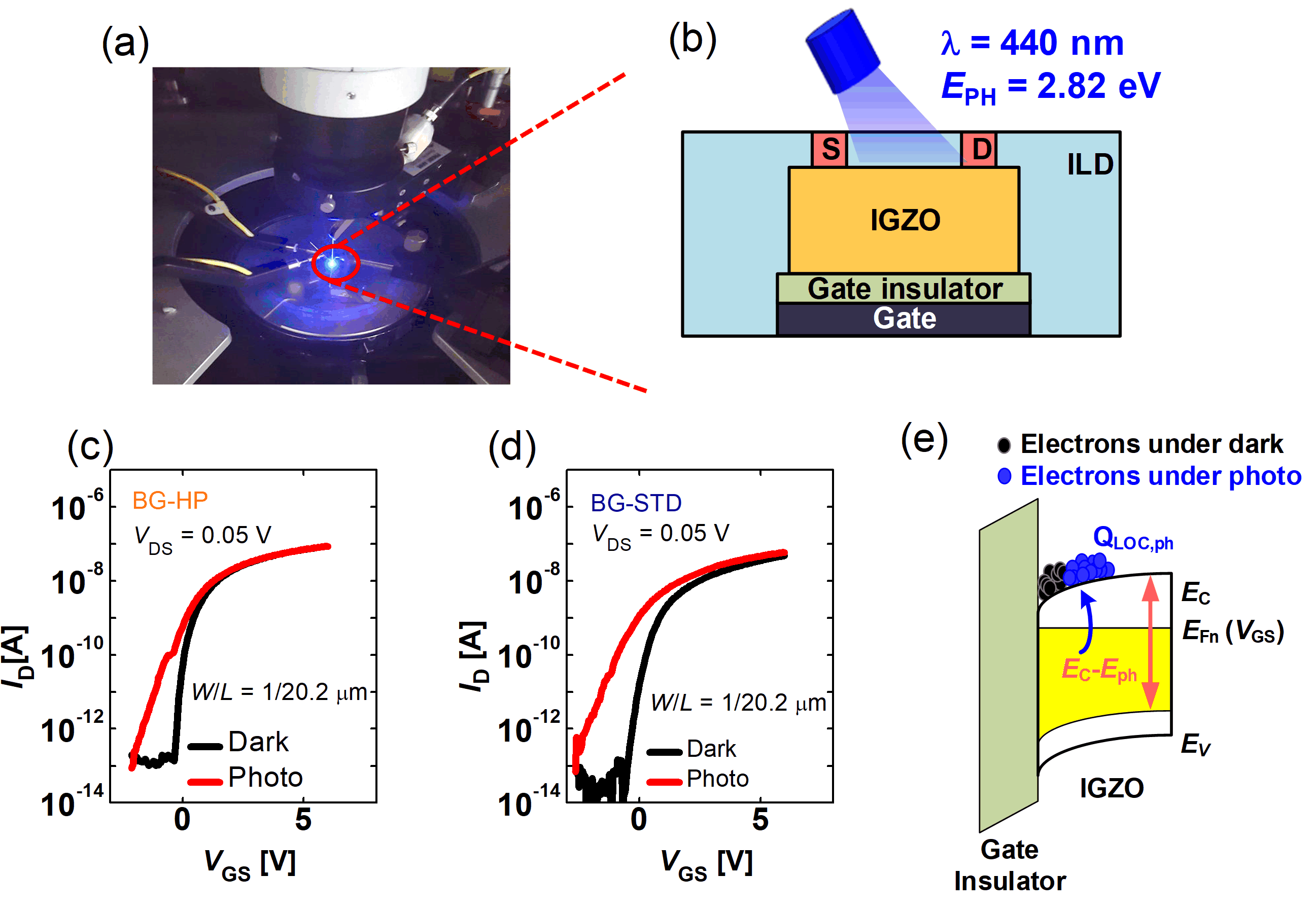
**

**Figure S4.** (a) A photograph and (b) schematic illustration for measuring the photo-response of the *I-V* characteristics of the IGZO FETs. The dark and photonic *I*D−*V*GS curves at *V*DS=0.05 V measured in (c) BG-HP and (d) BG-STD samples. (e) The energy band diagram under the photo-excitation.

The energy band diagram under the photo-excitation is illustrated in Fig. S4(e). When the drain current (*I*D) is measured in the dark state, the current is formed by the *V*GS-induced electrons under dark condition, but in a light illumination condition, the photo-excited carrier electrons are formed additionally, and the *I*D increases. The illumination-induced increase in *I*D originates from the number of electrons that are photo-excited from the localized states with the energy *E* range of *E*C−*E*ph < *E* < *E*Fn to the CB, where the *E*ph is the energy of an incident photon and the *E*Fn is the electron quasi-Fermi level. Then, the charge density formed by the photo-excited electrons (*Q*LOC,ph) is a function of *E*Fn(*V*GS) and *E*ph, and can be derived as follows.

$Q_{LOC,ph}\left(V_{GS}\right)=\frac{I_{ph}\left(V_{GS}\right)-I_{dark}\left(V_{GS}\right)}{\mu_{band}\times\frac{V_{DS}}{L}\times W\times t_{act}}$ (S1)

where the *I*ph and *I*dark are the photonic and dark current, band is the CB mobility, and *t*act is the thickness of the IGZO active film. Eq. (S1) suggests that the electrons pumped by light can drift by the electric field of the *V*DS, and the *Q*LOC,ph can be calculated by using the difference of free carrier concentration between in the dark and in the photo state. At this time, to calculate the DOS *g*(*E*), i.e., the trap concentration per unit energy and unit volume, one can use the following equation.

$g(E)=\frac{dQ_{LOC,ph}}{dV_{GS}}\times\frac{dV_{GS}}{dE_{Fn}}\times\frac{1}{q}$ (S2)

where the *q* is the magnitude of the elementary charge of a single electron, and the energy level *E* is scanned by modulating the *V*GS.

The term *dQ*LOC,ph/*dV*GS is explicitly a function of *V*GS, easily obtained through measurement and denoted by *g*(*V*GS). Then, to calculate the DOS [*g*(*E*)], a relationship between the *V*GS and the *E*Fn, i.e., *dV*GS/*dE*Fn, is required to be derived. The procedure of the energy-level mapping is described below.

A relationship between *V*GS and **S can be obtained as follows:

(S3)

where the *E*FB is the *E*C−*E*Fn at a flat band condition (*V*GS=*V*FB), and the **S is the surface potential in the channel of IGZO FET.

Fig. S5 shows the DOS extraction procedure based on the self-consistency between the photonic *I-V* and TCAD simulation. The *V*FB and the *V*GS-modulated **S are reflected in the relationship between *E*Fn−*E*C and *V*GS [Fig. S5(a)]. Here, the *V*FB is experimentally extracted27. In addition, *g*(*V*GS) is taken from the photonic *I-V* and Eq. (S1) and (S2) [Fig. S5(b)]. Then, in *V*GS > *V*FB condition, the *E*Fn(*V*GS) is easily obtained using Eq. (S3). However, in *V*GS < *V*FB condition (especially when *E*Fn is located near *E*V level), the *E*Fn(*V*GS) cannot be obtained only by Eq. (S3) due to the effect of back potential or floating body effect. Thus, for the energy level near *E*V, the *g*(*E*) extraction relies on the numerical iteration until satisfying the self-consistency between *g*(*V*GS) and *E*Fn(*V*GS) by adjusting *N*TD and *kT*TD [Fig. S5(c)].

**
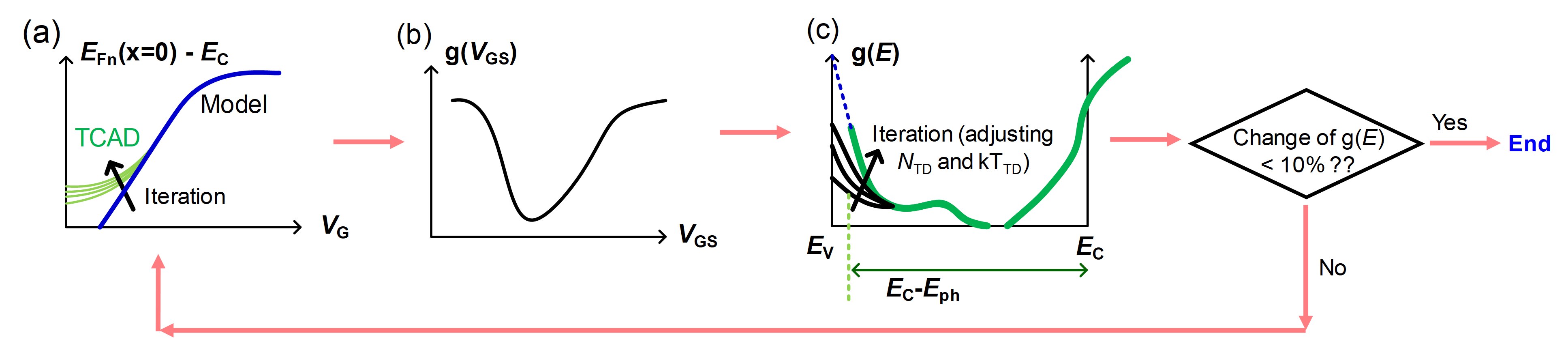
**

**Figure S5.** The DOS extraction procedure which is based on the self-consistency between the photonic *I-V* and TCAD simulation. (a) The relationship between *E*Fn−*E*C and *V*GS, which considers both the *V*FB and the *V*GS-modulated **S. The *E*Fn(*x*=0) means the *E*Fn at the surface of the front channel of FET. (b) Schematic illustration of the *g*(*V*GS) taken from *dQ*LOC,ph/*dV*GS. (c) Schematic view of *g*(*E*) reflecting the procedure of numerical iteration until satisfying the self-consistency between *g*(*V*GS) and *E*Fn(*V*GS) with the adjustment of *N*TD and *kT*TD.

In the priority of procedure, it is assumed that the *E*Fn(*V*GS) relationship in *V*GS > *V*FB condition, i.e., Eq. (S3), works well even for *V*GS < *V*FB condition [blue line in Fig. S5(a)]. Then, the *dV*GS/*dE*Fn is derived and combined with *g*(*V*GS); *g*(*E*) is constituted by using Eq. (S2). After that, the *E*Fn(*V*GS) is calculated from TCAD simulation based on the pre-assumed *g*(*E*). Then, the calculated *E*Fn(*V*GS) is compared with the assumed *E*Fn(*V*GS). The *N*TD and *kT*TD are adjusted until the self-consistency among *E*Fn(*V*GS), *V*FB, g(*V*GS), and *g*(*E*) is satisfied. In these fitting processes, the numerical iteration is allowed until the average error between the calculated and assumed values falls within a specific error rate (ER). The ER can be chosen considering the trade-off between the precision of the model and the computing burden of simulation. An ER = 10 % is used in this study.

Finally, extracted DOS parameters are summarized in Table S1.

**Model parameter extraction.** Fig. S6 shows the overall procedure of extracting TCAD model parameters. First of all, the structural parameters for the BG structure [Fig. 4 (a)] and the DG structure [Fig. 4 (b)] were assigned to TCAD frameworks, such as the *L* (*L*BG and/or *L*TG), IGZO thickness (*t*act), the separation between TG and S/D (*t*s), BG insulator thickness (*t*BGI), and TG insulator (*t*TGI). Material-related parameters were set as; the IGZO bandgap (*E*g_IGZO = 3eV)S1, the IGZO electron affinity (χIGZO = 4.3eV)S2, the IGZO permittivity (εIGZO = 11.7)S3, the Al2O3 permittivity (εAl2O3 = 9)S4, the SiO2 permittivity (εSiO2 = 3.9), the TiN work function (φTiN = 4.65 eV)S5, and the *p*+-Si Fermi level = 4.05 eV. Meanwhile, the DOS parameters were incorporated into the TCAD simulation framework, and the *m*n *and the *N*C were calculated through Eq. (4) and (5).


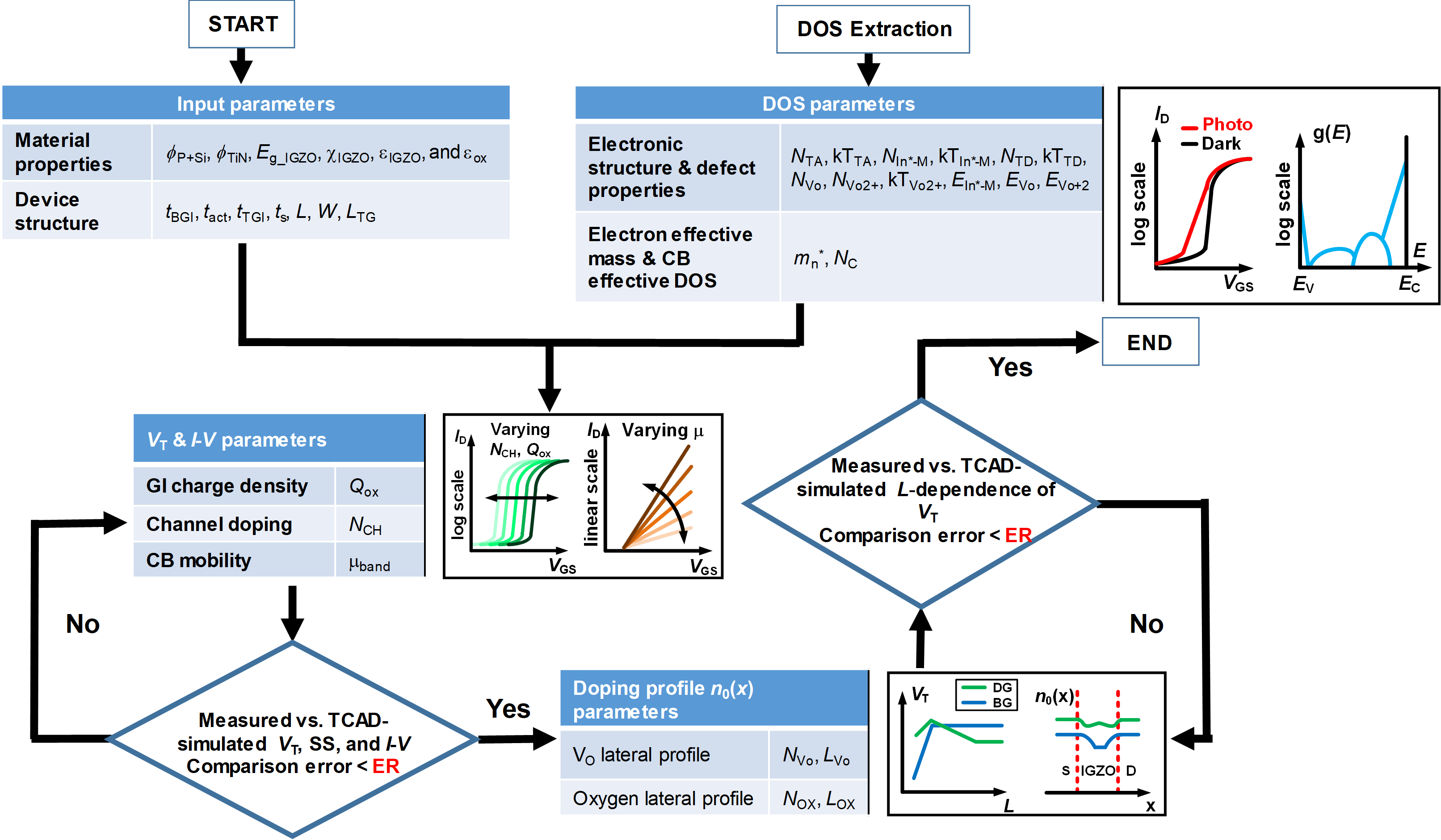


**Figure S6.** Overall TCAD model parameter-extracting procedure.

Next, the parameters, such as the channel doping concentration (*N*CH), the CB mobility (µband), the GI capacitance (*C*ox), and the oxide charge (*Q*ox), were extracted at the long *L* devices(*L*BG = 20.2 µm), where the diffusion effect of *V*O or O hardly affected the electrical properties in the S/D. At first, we can calculate the *V*FB as follows.

(S4)

Here, the *V*FB can be measured through the multi-frequency *C–V* methodS6, and the *E*FB (*E*C−*E*Fn in flat-band condition) is already known in the procedure of energy-level mapping in Fig. S5. The *Q*ox can be then extracted by Eq. (S4) through the measured *V*FB and *E*FB. Once *Q*ox is extracted, the *N*CH can be extracted by fitting with the measured *V*T since the *N*CH only remains as the *V*T-determining factor. Next, we determine the µband by fitting until the simulated ∂*I*D/∂*V*GS and measured ∂*I*D/∂*V*GS are matched. Then, the parameters (*N*Vo, *L*Vo, *N*OX, and *L*OX) determining the doping profile of the IGZO are extracted by comparing the measured *L*-dependency of *V*T with the simulated one, under the precondition of *N*OX = *N*CH_BG − *N*CH_DG.

Moreover, the parameters, such as µband, *Q*ox, and *C*ox, are updated at the short *L* devices (*L*BG = 0.5 µm) by the same procedure. For all cases, the used ER is 10 %.

Finally, all parameters are summarized in Table S1 as follows. They are validated by the self-consistency among the measured *I-V*, *V*T, *V*FB, SS, FE, DOS, and the *L*-dependency of *V*T, along with the four types of IGZO FETs (BG-STD, BG-HP, DG-STD, and DG-HP) and for both the long and short *L*.

**Table S1.** Extracted model parameters

|  | | |  | | | | | | |
| --- | --- | --- | --- | --- | --- | --- | --- | --- | --- |
| **Parameter**  **Definition** | | | **HP** | | **STD** | | | |  |
| **Material properties** | p+-Si / TiN / IGZO  (eV / eV / eV) | | 4.05 / 4.65 / 4.65 | | | | | |  |
|  | *E*g_IGZO / εIGZO / εox  (eV / - / -) | | 3 / 11.7ε0 / 3.9ε0 | | | | | |  |
| **Device structure** | *t*BGI / *t*act / *t*TGI  (nm / nm / nm) | | 6.5 / 12 (BG), 10 (DG) / 7 | | | | | |  |
|  | *W* / *L*BG  (μm / μm) | | 1 / 20.2 (long channel), 0.5 (short channel) | | | | | |  |
|  | *t*s / *L*TG  (μm / μm) | | 0.1 / 20 (long channel), 0.3 (short channel) | | | | | |  |
| **DOS** | *N*TA ­/ *kT*TA  (eV-1cm-3/ eV) | | 1.5×10^19^ **/** 0.09 | | | 2.2×10^19^**/** 0.09 | | |  |
|  | *N*In*-M / *kT*In*-M / *E*In*-M  (eV-1cm-3 / eV / eV) | | 3×10^13^ **/** 0.3 / 1 | | | 7×10^15^ / 0.4 / 1 | | |  |
|  | *N*TD / *kT*TD  (eV-1cm-3 / eV) | | 3×10^16^ / 0.03 | | | 1×10^17^ / 0.03 | | |  |
|  | *N*Vo / *kT*Vo / *E*Vo  (eV-1cm-3/ eV / eV) | | 8×10^11^ / 0.8 / 1.2 | | | 1×10^14^ / 0.5 / 1.2 | | |  |
|  | *N*Vo2+ / *kT*Vo2+ / *E*Vo2+  (eV-1cm-3/ eV / eV) | | 7×10^16^ / 0.3 / 0.2 | | | 2×10^17^ / 0.3 / 0.2 | | |  |
|  | *N*C / *m*n*  (cm-3 / kg) | | 2.57×10^17^/ 0.025*m*0 | | | 3.77×10^17^ / 0.041*m*0 | | |  |
|  | | | **BG-HP** | **DG-HP** | | **BG-STD** | **DG-STD** | Unit | |
| **Doping profile** | *N*CH | | 1016 | 1.4×10^18^ | | 4×10^17^ | 5×10^18^ | cm-3 | |
|  | *N*Vo | | 1018 | 2.5×10^18^ | | 1.4×10^18^ | 8×10^18^ | cm-3 | |
|  | *L*Vo | | 0.3 | 0.3 | | 0.5 | 0.35 | μm | |
|  | *N*OX | | - | 1.39×10^18^ | | - | 4.6×10^18^ | cm-3 | |
|  | *L*OX | | - | 0.5 | | - | 0.45 | μm | |
| band (*L* = 0.5 μm) | |  | 8 | 5 | | 9.5 | 5.5 | cm2V-1s-1 | |
| band (*L* = 20.2 μm) | |  | 9.5 | 23 | | 11 | 23 | cm2V-1s-1 | |
| *Q*ox | |  | −2×1012× *q* | | | | | | C/cm2 |
| *C*ox | |  | 5.31×10^-7^ | | | | | | F/cm2 |

**References (Supplementary information)**

S1. Kim, J. Bang, J., Nakamura, N. & Hosono, H. NBIS-stable oxide thin-film transistors using ultra-wide bandgap amorphous oxide semiconductors. *SID Symp. Dig. Tech. Papers*, 951-953 (2016).

S2. Lee, D. H., Nomura, K., Kamiya, T. & Hosono, H. Diffusion-limited a-IGZO/Pt Schottky junction fabricated at 200 °C on a flexible substrate. *IEEE Electron Device Lett* **32**, 1695-1697 (2011).

S3. Baek, G. & Kanicki, J. Modeling of current-voltage characteristics for double-gate a-IGZO TFTs and its application to AMLCDs. *Journal of the Society for Information Display* **20**, 237-44 (2012).

S4. Meza-Arroyo, J. *et al*. Ultra-dry air plasma treatment for enhancing the dielectric properties of Al2O3-GPTMS-PMMA hybrid dielectric gate layers in a-IGZO TFT applications. *Nanotechnology* **32**, 135203 (2021).

S5. Liu, Y. *et al*.Investigation of the TiN gate electrode with tunable work function and its application for FinFET fabrication. *IEEE Trans. Nanotechnol* **5,** 723-726 (2006).

S6. Choi, S. *et al*. Extraction Technique for Flat Band Voltage Using Multi-Frequency C−V Characteristics in Amorphous InGaZnO Thin-Film-Transistors*. IEEE Electron Device Letters* **41,** 1778-1781 (2020).
